# New Insights into Diarrhea Caused by High-Fat Diet and Fatigue: Gut Microbiota Dysbiosis-Driven Bile Acid Metabolism Disorder

**DOI:** 10.3390/nu18091317

**Published:** 2026-04-22

**Authors:** Qin Liu, Huiyi Peng, Xuejiao Xie, Miao Jiang, Maijiao Peng, Zhoujin Tan

**Affiliations:** School of Traditional Chinese Medicine, Hunan University of Chinese Medicine, Changsha 410208, China; liuqin998@163.com (Q.L.); anapenghuiyi@163.com (H.P.); xuejiao123@hnucm.edu.cn (X.X.); jiangmiao2211@163.com (M.J.)

**Keywords:** fatigue, high-fat diet, diarrhea, gut microbiota, bile acid metabolism, gut–liver axis

## Abstract

**Background:** This study investigated the mechanisms underlying diarrhea induced by a high-fat diet (HFD) under a state of fatigue, focusing on gut microbiota dysbiosis, bile acid metabolic disturbance, and gut–liver injury. **Methods:** Mice were assigned to a normal control diet (NCD) group, a HFD-induced diarrhea under fatigue (HFDM) group, and a HFD-induced diarrhea with aggravated dysbiosis (HFDMA) group. Histopathology, inflammatory factors, intestinal barrier-related proteins, small-intestinal microbiota, and colonic bile acid profiles were assessed, and correlation analyses were performed among gut microbiota, bile acids, and inflammatory factors. **Results:** Compared with the NCD group, both the HFDM and HFDMA groups showed diarrhea-like and fatigue-like phenotypes, histopathological injury in the small intestine and liver, increased tumor necrosis factor-alpha (TNF-α) and interleukin-6 (IL-6) levels, and impaired intestinal barrier function. No significant differences in inflammatory factors were observed between the HFDM and HFDMA groups. Zonula occludens-1 (ZO-1) expression decreased in both model groups but reached statistical significance only in the HFDMA group, whereas Claudin-1 expression was significantly reduced in both groups. Gut microbiota analysis showed altered community structure, with downward trends in alpha diversity that did not reach statistical significance but clear separation trends in beta diversity. Proteobacteria and *Streptococcus* increased, whereas *Ligilactobacillus* decreased. Total bile acid levels did not differ significantly among groups; however, the ratio of secondary to primary bile acids was significantly reduced in both model groups, particularly in the HFDMA group, with decreases in representative secondary bile acids, including hyodeoxycholic acid (HDCA) and isolithocholic acid (isoLCA). Correlation analysis further supported close associations among gut microbial alteration, bile acid disturbance, and intestinal and hepatic inflammation. **Conclusions:** Gut microbiota dysbiosis may disrupt bile acid metabolism, impair intestinal barrier integrity, and promote intestinal and hepatic inflammatory responses, thereby contributing to diarrhea progression under fatigue and HFD conditions through the gut–liver axis.

## 1. Introduction

Diarrhea, a prevalent gastrointestinal disorder in clinical settings, is principally defined by increased fecal water content, higher bowel movement frequency, and alterations in stool form. With approximately 1.17 million annual deaths globally, diarrhea represents a substantial threat to public health [1]. Children under five are among the most vulnerable populations, and older adults, particularly those aged 70 and over, also experience a substantial burden of mortality [2]. The etiology of diarrhea is multifactorial, encompassing factors such as fatigue, dietary patterns (especially high-fat intake), environmental conditions (such as high humidity), genetic predisposition, and psychological stress. Prior research indicates that fatigue and a high-fat diet (HFD) can trigger diarrhea [3,4,5]. Importantly, high-fat consumption may worsen diarrhea in susceptible individuals, especially those with bile acid diarrhea (BAD), chronic functional diarrhea, or diarrhea-predominant irritable bowel syndrome (IBS-D) [6,7]. From the perspective of the gut–liver axis, HFD may contribute to diarrhea by inducing gut microbiota dysbiosis, disturbing bile acid homeostasis, and impairing intestinal barrier function, thereby promoting the translocation of microbial products and exacerbating inflammatory responses along the intestine–liver axis [8,9]. In this context, bile acids are increasingly recognized as key mediators linking dietary fat exposure, microbial dysbiosis, mucosal barrier dysfunction, and host inflammatory responses [8,9]. Nevertheless, the pathophysiological mechanisms by which fatigue and HFD-induced diarrhea remain incompletely understood.

In recent years, the gut microbiota has drawn considerable attention for its critical role in intestinal homeostasis and is often described as the “second genome” [10]. Our team’s previous studies using a mouse model of diarrhea induced by fatigue combined with a HFD demonstrated marked alterations in gut microbiota composition, including an increased abundance of Lactobacillus murinus and decreased abundances of Lactobacillus intestinalis, Lactobacillus johnsonii, and Lactobacillus reuteri [3,4,5]. Moreover, intestinal barrier dysfunction in this model appeared to be closely associated with reduced Lactobacillus reuteri abundance and enhanced intestinal inflammatory responses [11]. Additional studies using a mouse model of diarrhea induced by cold drink combined with a HFD further showed that the abundances of specific bacterial groups, including norank_f_Muribaculaceae, Muribaculum, and Odoribacter, were markedly decreased, accompanied by downregulation of the tight junction proteins zonula occludens-1 (ZO-1) and Claudin-1 and increased levels of the pro-inflammatory cytokines interleukin-6 (IL-6) and tumor necrosis factor-alpha (TNF-α) [12]. In contrast, fecal microbiota transplantation has proven to be efficacious in ameliorating diarrhea in young cattle and reinstating the structural integrity of the gut microbiota [13]. Collectively, these data suggest that gut microbiota dysbiosis affects the development of diarrhea via various pathways, such as compromising the integrity of the intestinal barrier and stimulating the release of inflammatory mediators. Nevertheless, the precise molecular mechanisms involved remain to be thoroughly elucidated.

Emerging studies suggest that gut microbiota–host interactions along the gut–liver axis are key contributors to the development and progression of diarrhea [14]. As a key element of this axis, bile acid metabolism is closely associated with the advancement of diarrhea. Research indicates that the proportion of primary bile acids in the feces of patients with diarrhea is markedly increased, a phenomenon potentially attributable to an insufficiency in secondary bile acid conversion resulting from gut microbiota dysbiosis [15]. In a rat model of chronic fatigue syndrome, alterations in the gut microbiota, characterized by an increased abundance of *Proteobacteria* and a decreased abundance of *Bacteroidetes* and the genus *Lactobacillus*, are accompanied by elevated levels of primary bile acids [16]. Furthermore, studies have demonstrated that a HFD can promote the overgrowth of *Bilophila wadsworthia* by increasing TCA, thereby intensifying colonic inflammation in rats. Conversely, *Lactobacillus reuteri* mitigates bile acid-induced diarrhea by inhibiting the glucuronidation of bile acids through its metabolic product, indole-3-methanol [17].

Based on the gut–liver axis theory, this study systematically explored the interactions among gut microbiota, bile acid metabolism, and diarrhea. Unlike many previous studies that focused on fecal or colonic microbiota, the present study used small-intestinal contents for microbiota profiling, as the small intestine is the primary site of nutrient digestion and absorption and plays a key role in host–microbe interactions, mucosal immunity, barrier integrity, and bile acid homeostasis [18]. In contrast, colonic contents were selected for bile acid metabolomic analysis because most bile acids are reabsorbed in the distal ileum, whereas those entering the colon undergo further microbiota-mediated transformation, especially into secondary bile acids [19]. By combining microbiota profiling with bile acid metabolomics, this study aimed to provide experimental evidence and a theoretical basis for targeting the gut microbiota–bile acid axis in the treatment of diarrhea.

## 2. Materials and Methods

### 2.1. Dietary Components

The experimental basal diet was formulated to contain at least 20% crude protein, 4% crude fat, and 1.3% lysine. Meanwhile, the levels of crude fiber, crude ash, and moisture were controlled below 5%, 8%, and 10%, respectively, and the contents of calcium, phosphorus, and sodium chloride were maintained within the ranges of 0.6–1.8%, 0.6–1.2%, and 0.3–0.8%. According to the product nutrition label, per 100 g of the lard used for the high-fat intervention contained 100.0 g fat and provided 3700 kJ energy, corresponding to 167% of the nutrient reference value (NRV) for fat and 44% of the NRV for energy, respectively. The lard was stored at room temperature and heated to 37 °C before use, and administered to mice by gavage.

### 2.2. Drugs and Reagents

A mixed antibiotic solution was freshly prepared by dissolving gentamicin sulfate injection and cefradine capsules in sterile water to a final concentration of 62.5 g/L and was used immediately after preparation [13]. Gentamicin sulfate injection (2 mL: 80 mg; batch No. 3C3030612) was purchased from Yichang Renfu Pharmaceutical Co., Ltd., Jining, China, and cefradine capsules (0.25 g; batch No. 53241012) were obtained from Shandong Lukang Pharmaceutical Co., Ltd., Jining, China. Lard (catalog No. SC1033713020099) was supplied by Linyi Xincheng Jinluo Meat Products Group Co., Ltd., Linyi, China. The mouse tumor necrosis factor-alpha (TNF-α) enzyme-linked immunosorbent assay (ELISA) kit (batch No. 202506; catalog No. JM-02415M1) and interleukin-6 (IL-6) ELISA kit (batch No. 202506; catalog No. JM-02446M1) were purchased from Jiangsu Jingmei Biotechnology Co., Ltd., Nanjing, China.

### 2.3. Animals

Thirty specific pathogen-free (SPF) male Kunming mice, aged 4 weeks and weighing 20 ± 2 g, were obtained from Hunan SJA Laboratory Animal Co., Ltd., Changsha, China (license No. SCXK [Xiang] 2021-0002). Animals were maintained under standard SPF conditions at 23–25 °C with 50–70% relative humidity and a 12 h light/dark cycle. Five mice were housed per cage, giving a total of six cages, with two cages assigned to each experimental group. All animal procedures were approved by the Animal Ethics Committee of Hunan University of Chinese Medicine (Approval No. HNUCM21-2025-10).

### 2.4. Experimental Design

**Figure 1:**
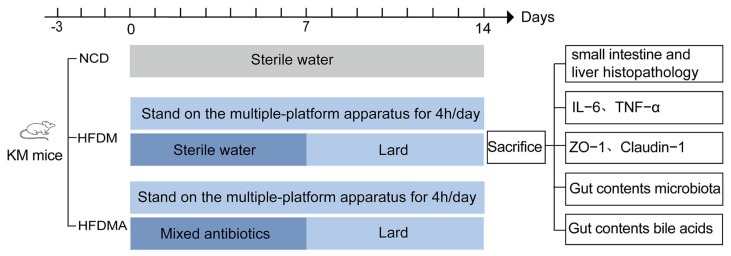
The flowchart of the experimental protocol.

Mice were randomly divided into three groups: the normal control group (NCD, *n* = 10), the HFD-induced diarrhea model group (HFDM, *n* = 10), and the aggravated model group with antibiotic-induced gut microbiota dysbiosis (HFDMA, *n* = 10). To induce diarrhea, mice in the HFDM and HFDMA groups were exposed to multi-platform stress for 4 h per day over 14 consecutive days. In addition, from day 8 to day 14, both groups received lard emulsified in hot water by intragastric gavage twice daily at 20 mL/(kg·d) [4]. To further exacerbate diarrhea through disruption of the gut microbiota, mice in the HFDMA group were additionally given an antibiotic mixture by intragastric gavage twice daily at 23.33 mL/(kg·d) from day 1 to day 7 [20]. Thus, compared with the HFDM group, the HFDMA group underwent the same stress and lard interventions, but with additional antibiotic pretreatment during the first 7 days. In contrast, mice in the NCD group were maintained under normal conditions and received sterile water by gavage twice daily throughout the 14-day experimental period. After model establishment, the open-field test was conducted, and the mice were subsequently euthanized by cervical dislocation. Intestinal contents, intestinal tissues, and liver tissues were then collected for further analysis. The experimental design is illustrated in Figure 1.

### 2.5. General Condition and Fecal Water Content of Mice

The general condition of the mice, including their mental status and fecal characteristics, was observed. Fresh fecal samples were weighed to determine wet weight and then dried at 110 °C to constant weight for measurement of dry weight. Fecal water content was calculated as follows: fecal water content (%) = (wet weight − dry weight)/wet weight × 100%.

### 2.6. Open-Field Test

Following model establishment, five mice were randomly chosen from each group and evaluated in the open-field test using the KSYY-OP-V4.0 real-time monitoring and analysis system. Mice were individually placed in the arena for 300 s, and total distance traveled, average speed, and immobility time were recorded automatically. The test was conducted under lights-off conditions to simulate the animals’ active period. The arena was cleaned with 75% ethanol after each trial to avoid interference between animals.

### 2.7. Hematoxylin and Eosin Staining (H&E)

Small intestine and liver tissues were fixed, paraffin-embedded, and sectioned for histopathological evaluation. After baking, the sections were deparaffinized in a graded manner and then stained with hematoxylin and eosin.

### 2.8. Enzyme-Linked Immunosorbent Assay (ELISA)

TNF-α and IL-6 in the small intestine and liver were assayed using commercial ELISA kits in accordance with the manufacturers’ instructions, and their concentrations were calculated from the optical density (OD) readings.

### 2.9. Immunohistochemistry (IHC)

IHC staining was performed on paraffin-embedded small-intestinal sections to detect ZO-1 (ab221547, Abcam, Cambridge, UK; 1:400) and Claudin-1 (13050-1-AP, Proteintech, Chicago, IL, USA; 1:400). Following antigen retrieval and blocking, the sections were incubated with primary antibodies at 4 °C overnight and then with an HRP-linked goat anti-rabbit secondary antibody (ab205718, Abcam; 1:2000). Signals were visualized using DAB, and nuclei were counterstained with hematoxylin. Images were captured under a Nikon Eclipse CI microscope (Nikon Corporation, Tokyo, Japan), and AOD was quantified with FIJI Image software (version 1.53c, National Institutes of Health, Bethesda, MD, USA).

### 2.10. 16S Ribosomal RNA (16S rRNA) Gene Sequencing and Bioinformatics Analysis

Because the small intestine is a major site of lipid digestion, nutrient absorption, and mucosal immune activity, small-intestinal contents were selected for microbiota analysis. Under aseptic conditions, the abdominal cavity was opened, the small intestine was isolated, and the luminal contents were collected. 16S rRNA gene sequencing was performed as previously described [21]. Total nucleic acids were extracted from homogenized small-intestinal contents using the Magbeads FastDNA Kit for Soil (Cat. No. 116564384, MP Biomedicals, Irvine, CA, USA), and DNA concentration was measured using a NanoDrop spectrophotometer (Thermo Fisher Scientific, Waltham, MA, USA). The V3–V4 region of the bacterial 16S rRNA gene was amplified using primers 338F and 806R. Amplicons were quantified with the Quant-iT PicoGreen dsDNA Assay Kit (BioTek, FLx800, Inc., Winooski, VT, USA), pooled in equimolar amounts, and used for library construction with the Illumina TruSeq Nano DNA LT Library Prep Kit (Thermo Fisher Scientific, Waltham, MA, USA). Paired-end sequencing (2 × 250 bp) was performed on the Illumina NovaSeq platform using the NovaSeq 6000 SP Reagent Kit (500 cycles, Illumina, Inc., San Diego, CA, USA). Paired-end reads were first processed using q2-cutadapt for primer removal and then analyzed with the DADA2 denoise-paired workflow in QIIME2, which performs quality filtering, denoising, paired-end merging, and chimera removal. To avoid ambiguity, sequencing statistics are reported as input reads, filtered reads, denoised reads, merged reads, and non-chimeric reads, rather than as “effective reads”. Detailed per-sample sequencing statistics, including Q20, Q30, GC content, and merged-read counts, are provided in Supplementary Material S1. Because this study used targeted 16S rRNA amplicon sequencing of luminal small-intestinal contents rather than shotgun metagenomic sequencing of host-rich tissue samples, host-versus-bacterial genome mapping rates were not generated as part of the standard workflow. After quality control and taxonomic assignment, only bacterial ASVs were retained for downstream analysis. ASVs were assigned taxonomically against the Greengenes 2 database, and downstream analyses were performed using R (version 4.2.0, R Foundation for Statistical Computing, Vienna, Austria) and QIIME2 (version 2022.2). Sequencing services were provided by Shanghai Personal Biotechnology Co., Ltd., Shanghai, China.

### 2.11. Targeted Profiling of Bile Acids Based on Ultra-High-Performance Liquid Chromatography–Tandem Mass Spectrometry (UHPLC-MS/MS) Analysis

#### 2.11.1. Method Validation and Quality Control

Method validation included precision, accuracy, recovery, stability, and quality control (QC). Precision, accuracy, and recovery were evaluated at three concentration levels over three consecutive days. Acceptance criteria were defined as RSD ≤ 15% for precision and recovery within 85–115% with RSD ≤ 15% for accuracy and recovery [22,23]. Stability was assessed after storage in the autosampler at 4 °C for 24 h, with RSD ≤ 15% considered acceptable [23]. In addition, QC samples were analyzed periodically throughout each batch to monitor instrumental stability and analytical reproducibility. Detailed validation data are provided in Supplementary Material S2.

#### 2.11.2. Metabolite Extraction

Colonic content samples were diluted with water and vortexed thoroughly. Then, 20 μL of each diluted sample was mixed with 60 μL of acetonitrile/methanol (8:2, *v*/*v*) containing mixed internal standards, followed by sonication for 10 min, incubation at −20 °C for 60 min, and centrifugation at 12,000 rpm for 10 min. The resulting supernatants were collected for UHPLC-MS/MS analysis.

#### 2.11.3. UHPLC-MS/MS Analysis

Targeted bile acid quantification was performed by Novogene Co., Ltd. (Beijing, China) using an ExionLC™ AD UHPLC system coupled with a QTRAP 6500+ mass spectrometer (AB SCIEX Corp., Boston, MA, USA). Chromatographic separation was achieved on a Waters ACQUITY UPLC BEH C18 column (2.1 × 100 mm, 1.7 μm, Waters Corporation, Milford, MA, USA) maintained at 50 °C. The mobile phases consisted of water containing 0.1% formic acid and 5 mM ammonium acetate (A) and acetonitrile (B), delivered at a flow rate of 0.35 mL/min. The gradient elution program was as follows: 0–0.5 min, 5% B; 0.5–1.5 min, 5–30% B; 1.5–4.0 min, 30–37% B; 4.0–5.0 min, 37–38% B; 5.0–5.5 min, 38–39% B; 5.5–6.0 min, 39–42% B; 6.0–6.5 min, 42–43% B; 6.5–9.5 min, 43–50% B; 9.5–11.0 min, 50–60% B; 11.0–12.0 min, 60–95% B; 12.0–13.1 min, 95–5% B; and 13.1–15.0 min, 5% B. Mass spectrometric detection was conducted in negative multiple reaction monitoring mode under the following conditions: IonSpray Voltage, −4500 V; Curtain Gas, 30 psi; source temperature, 550 °C; and Ion Source Gas 1 and Gas 2, 60 psi.

#### 2.11.4. Standard Curves and Limit of Quantification

Calibration curves were generated using serial standard solutions by plotting analyte concentration against the peak area ratio of analyte to internal standard. Analytes with a correlation coefficient (r) > 0.99 were considered acceptable. The limit of quantification (LOQ) was defined as the concentration corresponding to a signal-to-noise ratio of 10:1 [22,23].

### 2.12. Correlation Analysis Method

Spearman’s correlation analysis was conducted to assess the associations among gut microbiota, differential bile acids, and inflammatory factors (TNF-α and IL-6). Furthermore, correlations between key bacterial taxa and bile acids, as well as between these variables and inflammatory markers in the small intestine and liver, were analyzed.

### 2.13. Statistical Analysis

Statistical analyses were carried out using IBM SPSS 25.0 and GraphPad Prism (version 10.0). Data are presented as mean ± standard deviation (M ± SD). One-way ANOVA was used for normally distributed data with equal variances, while nonparametric rank-sum testing was applied to data that did not meet these criteria. A value of *p* < 0.05 was considered statistically significant.

## 3. Results

### 3.1. General Conditions of Mice

**Figure 2:**
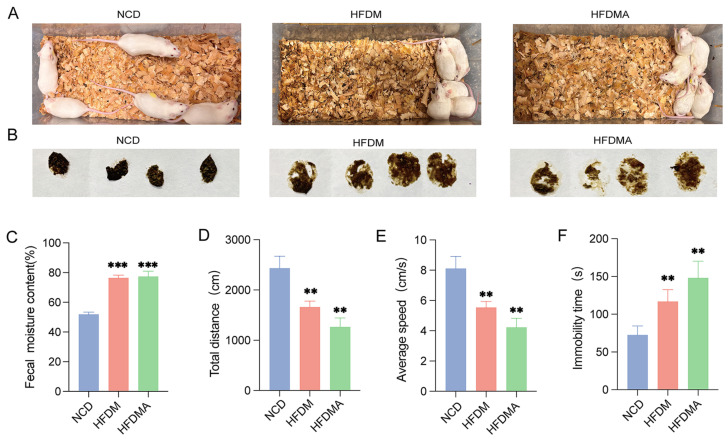
General condition and open-field performance of mice in each group. (**A**) Mental state and bedding condition. (**B**) Fecal morphology. (**C**) Fecal water content. (**D**) Total distance traveled. (**E**) Average speed. (**F**) Immobility time. Data are presented as mean ± SD (*n* = 5–8), and statistical significance was evaluated using one-way ANOVA. Compared with the NCD group, ** *p* < 0.01, *** *p* < 0.001. NCD: normal control group; HFDM: HFD- and fatigue-induced diarrhea model group; HFDMA: aggravated diarrhea model group with gut microbiota dysbiosis.

Mice in the NCD group maintained a normal general condition, with dry bedding and well-formed feces, whereas mice in the HFDM and HFDMA groups exhibited reduced activity, huddling behavior, wet bedding, and loose, unformed feces (Figure 2A,B). Compared with the NCD group, fecal water content was significantly increased in both the HFDM and HFDMA groups (*p* < 0.01; Figure 2C). Open-field test results further showed that total distance traveled and average speed were significantly decreased, whereas immobility time was significantly increased, in both the HFDM and HFDMA groups relative to the NCD group (all *p* < 0.01; Figure 2D–F). These findings support the successful establishment of a diarrhea model induced by HFD and fatigue in mice.

### 3.2. Histopathological Injury and Inflammatory Responses in the Small Intestine and Liver of Mice

**Figure 3:**
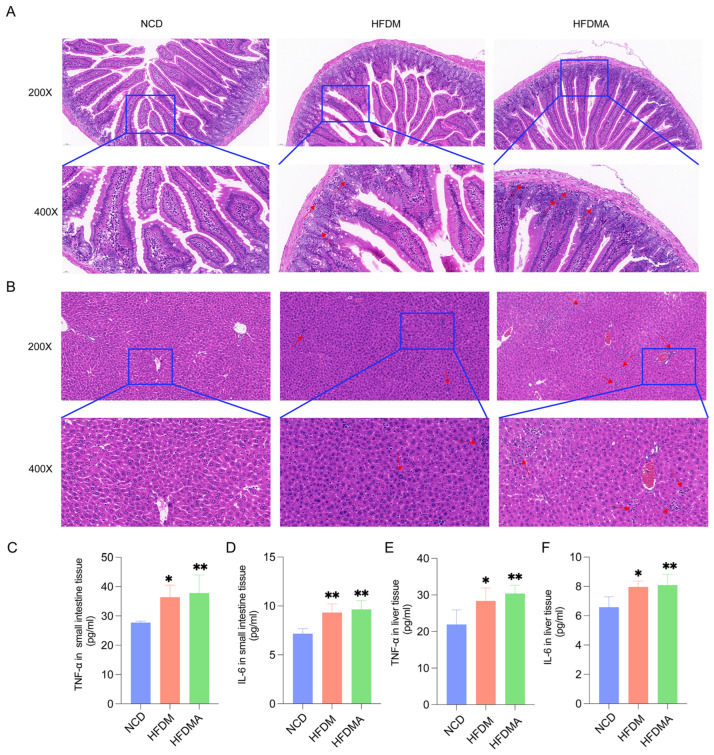
Histopathological changes and inflammatory factor levels in the small intestine and liver of mice in each group. (**A**) Representative H&E staining of small intestinal tissue (upper panel, 200×; lower panel, 400×). Scale bars: 50 µm and 20 µm, respectively. (**B**) Representative H&E staining of liver tissue (upper panel, 200×; lower panel, 400×). Scale bars: 50 µm and 20 µm, respectively. (**C**) TNF-α level in small intestinal tissue. (**D**) IL-6 level in small intestinal tissue. (**E**) TNF-α level in liver tissue. (**F**) IL-6 level in liver tissue. Red arrows indicate histopathological injury and inflammatory cell infiltration. Data are presented as mean ± SD (*n* = 5), and statistical significance was evaluated using one-way ANOVA. Compared with the NCD group, * *p* < 0.05, ** *p* < 0.01. NCD: normal control group; HFDM: HFD- and fatigue-induced diarrhea model group; HFDMA: aggravated diarrhea model group with gut microbiota dysbiosis.

H&E staining results are shown in Figure 3A,B, and inflammatory factor levels are presented in Figure 3C–F. The NCD group exhibited normal histological structures in both the small intestine and liver. In contrast, the HFDM group showed evident intestinal villous disorganization, crypt irregularity, mild inflammatory cell infiltration, and scattered inflammatory infiltration in the liver. These histopathological alterations were more pronounced in the HFDMA group, which exhibited more severe villous damage, greater inflammatory cell infiltration, and focal disturbance of hepatic structure (Figure 3A,B).

Consistent with these histopathological findings, TNF-α and IL-6 levels in both the small intestine and liver were significantly elevated in the HFDM and HFDMA groups compared with the NCD group (Figure 3C–F, *p* < 0.05 or *p* < 0.01). No significant differences were observed between the HFDM and HFDMA groups in either tissue, although the HFDMA group showed a trend toward higher inflammatory factor levels. These findings indicate that fatigue combined with a HFD-induced marked tissue injury and inflammatory responses in the small intestine and liver of mice.

### 3.3. Expression of ZO-1 and Claudin-1 in Small Intestine Tissue in Mice

**Figure 4:**
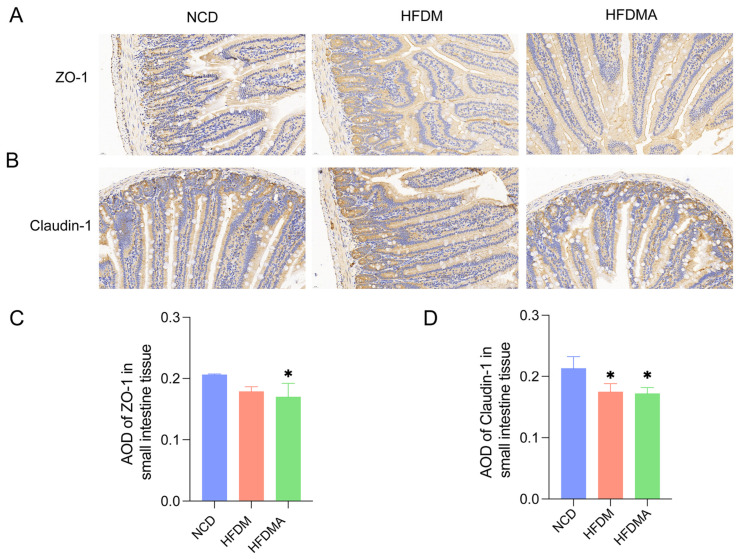
(**A**,**B**) Representative immunohistochemical staining of ZO-1 and Claudin-1 in small intestinal tissue. (**C**,**D**) Quantitative analysis of the average optical density (AOD) of ZO-1 and Claudin-1 in small intestinal tissue (*n* = 3). Data are presented as mean ± SD. Compared with the NCD group, * *p* < 0.05. NCD: normal control group; HFDM: HFD- and fatigue-induced diarrhea model group; HFDMA: aggravated diarrhea model group with gut microbiota dysbiosis.

Figure 4 shows reduced expression of small-intestinal barrier proteins in the model groups. ZO-1 expression declined in both the HFDM and HFDMA groups, although significance was reached only in the HFDMA group versus the NCD group (*p* < 0.05). Claudin-1 was significantly decreased in both model groups (both *p* < 0.05). No significant difference was observed between the HFDM and HFDMA groups for either ZO-1 or Claudin-1 (*p* > 0.05). Overall, these results suggest that fatigue combined with HFD disrupted small-intestinal barrier function in mice.

### 3.4. Changes in Species Composition of Intestinal Microbiota in Mice

**Figure 5:**
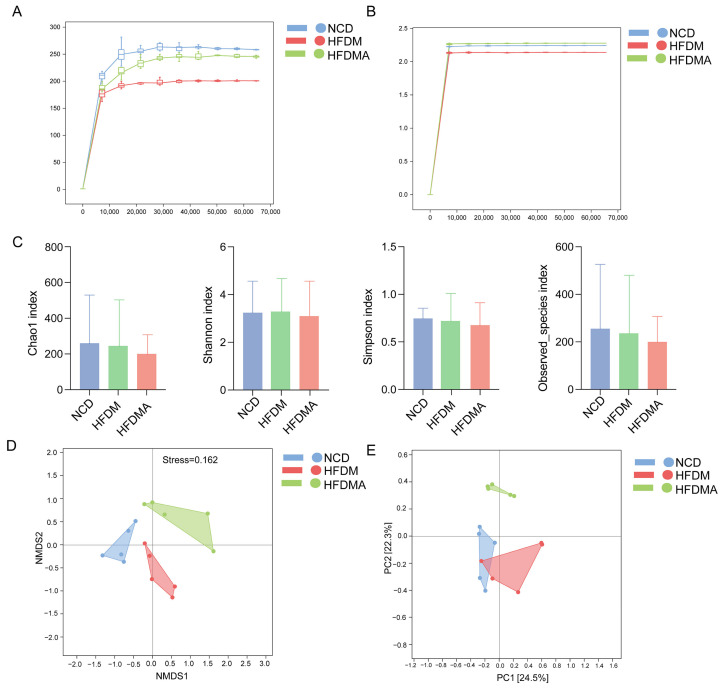
Alpha- and beta-diversity analyses of the small-intestinal microbiota. (**A**,**B**) Chao1 and Shannon rarefaction curves; (**C**) alpha-diversity indices; (**D**,**E**) NMDS and PCoA plots. Data are expressed as mean ± SD (*n* = 5), and statistical significance was evaluated using one-way ANOVA. NCD: normal control group; HFDM: HFD- and fatigue-induced diarrhea model group; HFDMA: aggravated diarrhea model group with gut microbiota dysbiosis.

As shown in Figure 5A,B, the Chao1 and Shannon rarefaction curves gradually approached saturation with increasing sequencing depth, indicating that the sequencing depth was sufficient to reflect microbial richness and diversity in the samples. Alpha diversity was assessed using the Chao1 and Observed species indices for richness and the Shannon and Simpson indices for diversity. Compared with the NCD group, both the HFDM and HFDMA groups showed lower Chao1, Simpson, and Observed species values, whereas the Shannon index showed no obvious change. However, none of these differences were statistically significant (Figure 5C). Overall, the model groups showed a downward trend in richness-related indices without significant alterations in alpha diversity.

Beta diversity analysis showed a tendency toward group-wise clustering. In the NMDS plot (Figure 5D), the stress value was 0.162, indicating acceptable model fit. In the PCoA plot (Figure 5E), PC1 and PC2 explained 24.5% and 22.3% of the variation, respectively. The HFDMA group was more clearly separated from the NCD and HFDM groups, although some overlap remained between the NCD and HFDM groups. These results suggest that fatigue combined with a HFD altered the structure of the small-intestinal microbiota.

**Figure 6:**
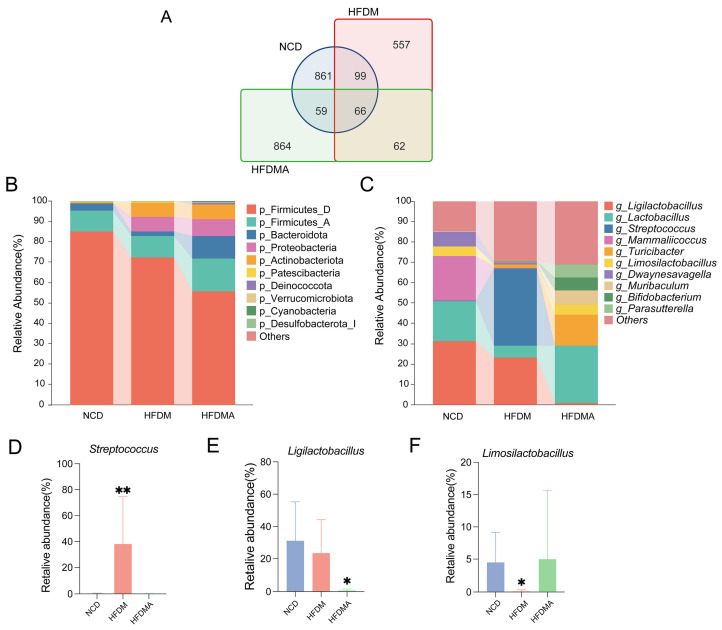
Gut microbiota composition in different groups. (**A**) Venn diagram of shared and unique ASVs among the NCD, HFDM, and HFDMA groups. The blue circles represent the NCD group, the red rectangles represent the HFDM group, and the green rectangles represent the HFDMA group. (**B**) Relative abundance of the top ten phyla. (**C**) Relative abundance of the top ten genera. (**D**–**F**) Group comparisons of selected genera. Data are expressed as mean ± SD (*n* = 5), and statistical significance was evaluated using one-way ANOVA. Compared with the NCD group, * *p* < 0.05, ** *p* < 0.01. NCD: normal control group; HFDM: HFD- and fatigue-induced diarrhea model group; HFDMA: aggravated diarrhea model group with gut microbiota dysbiosis. Taxa with suffixes such as _A and _B indicate database-defined phylogenetic clades in the Greengenes2/GTDB taxonomy.

As shown in Figure 6A, the numbers of detected ASVs in the NCD, HFDM, and HFDMA groups were 1085, 784, and 1051, respectively, with 861, 557, and 864 unique ASVs in each group. Meanwhile, 99, 59, and 62 ASVs were shared between the NCD and HFDM, NCD and HFDMA, and HFDM and HFDMA, respectively, and 66 ASVs were common to all three groups.

At the phylum level (Figure 6B), Firmicutes_D and Firmicutes_A were dominant in all groups, with Bacteroidota enriched in the NCD and HFDMA groups and Proteobacteria enriched in the HFDM group. At the genus level (Figure 6C), *Ligilactobacillus*, *Mammaliicoccus*, and *Lactobacillus* were dominant in the NCD group, whereas *Streptococcus* markedly increased in the HFDM group. The HFDMA group showed relatively high abundances of *Lactobacillus*, *Turicibacter*, *Muribaculum*, *Parasutterella*, and *Bifidobacterium*.

As shown in Figure 6D–F, Firmicutes_D tended to decrease in both the HFDM and HFDMA groups, whereas Proteobacteria increased relative to the NCD group. Bacteroidota was reduced in the HFDM group but relatively increased in the HFDMA group. At the genus level, *Streptococcus* was significantly elevated and *Limosilactobacillus* was significantly reduced in the HFDM group. *Ligilactobacillus* and *Lactobacillus* also decreased in the HFDM group. In the HFDMA group, *Ligilactobacillus* remained significantly reduced, while *Limosilactobacillus*, *Lactobacillus*, and *Bifidobacterium* showed relatively higher abundances than in the HFDM group.

### 3.5. Changes in Characteristic Gut Microbiota Taxa and Their Predicted Functions in the Gut Contents of Mice

**Figure 7:**
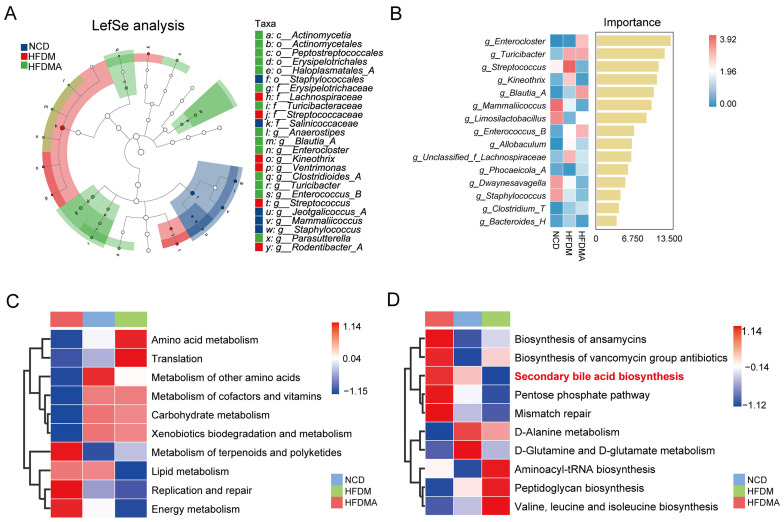
Changes in characteristic taxa and predicted functions of the small-intestinal microbiota in mice. (**A**) LEfSe analysis showing differentially enriched taxa among groups. (**B**) Random forest analysis of representative genera contributing to group discrimination. (**C**) Heatmap of representative KEGG level 2 functional categories predicted by PICRUSt2. (**D**) Heatmap of representative KEGG level 3 pathways predicted by PICRUSt2. NCD: normal control group; HFDM: HFD- and fatigue-induced diarrhea model group; HFDMA: aggravated diarrhea model group with gut microbiota dysbiosis.

As shown in Figure 7A, linear discriminant analysis effect size (LEfSe) analysis with a linear discriminant analysis (LDA) threshold of >4 identified differentially enriched taxa among the three groups. At the genus level, the NCD group was enriched in *Jeotgalicoccus_A*, *Mammaliicoccus*, and *Staphylococcus*; the HFDM group was enriched in *Kineothrix*, *Ventrimonas*, *Streptococcus*, and *Rodentibacter_A*; and the HFDMA group was enriched in *Anaerostipes*, *Blautia_A*, *Enterocloster*, *Clostridioides_A*, *Turicibacter*, *Enterococcus_B*, and *Parasutterella*. To further identify representative taxa contributing to group discrimination, random forest analysis was performed at the genus level (Figure 7B). The top-ranked genera included *Enterocloster*, *Turicibacter*, *Streptococcus*, *Kineothrix*, *Blautia_A*, *Mammaliicoccus*, *Limosilactobacillus*, and *Enterococcus_B*, suggesting that these taxa were among the major contributors to intergroup separation.

Phylogenetic Investigation of Communities by Reconstruction of Unobserved States 2 (PICRUSt2)-based functional prediction further revealed group-dependent differences in microbial metabolic potential (Figure 7C,D). KEGG level 2 analysis showed distinct functional enrichment patterns among groups. The HFDM group was characterized by relatively higher enrichment in amino acid metabolism and translation, whereas the HFDMA group exhibited greater enrichment in terpenoid and polyketide metabolism, replication and repair, and energy metabolism. In contrast, pathways related to cofactor and vitamin metabolism, carbohydrate metabolism, and xenobiotics biodegradation and metabolism were more abundant in the NCD and HFDM groups than in the HFDMA group, and the NCD group showed relatively higher enrichment in other amino acid metabolism. At KEGG level 3, biosynthesis of ansamycins, biosynthesis of vancomycin group antibiotics, secondary bile acid biosynthesis, the pentose phosphate pathway, and mismatch repair showed relatively higher predicted enrichment in the HFDMA group and lower predicted abundance in the HFDM group. In contrast, aminoacyl-tRNA biosynthesis, peptidoglycan biosynthesis, and valine, leucine and isoleucine biosynthesis appeared relatively enriched in the HFDM group. These results indicate that alterations in gut microbial composition were accompanied by corresponding changes in predicted metabolic functions.

### 3.6. Changes in Bile Acid Profiles and Differential Bile Acids in Mice

**Figure 8:**
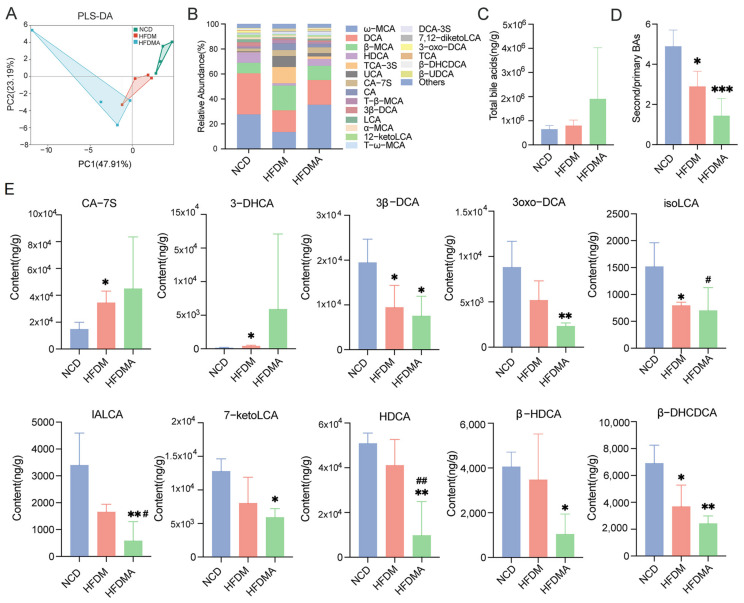
Bile acid profiling and differential bile acid analysis in mice. (**A**) PLS-DA score plot. (**B**) Relative composition of bile acid profiles. (**C**) Total bile acid levels. (**D**) Ratio of secondary to primary bile acids. (**E**) Differential bile acids among groups. Data are presented as mean ± SD. Panels (**C**,**D**) were analyzed using one-way ANOVA for normally distributed data with equal variances, or nonparametric rank-sum testing when these assumptions were not met. Differential bile acids in panel (**E**) were screened by pairwise univariate analysis using fold change (FC) > 1.2 and *p* < 0.05 as thresholds. NCD: normal control group; HFDM: HFD- and fatigue-induced diarrhea model group; HFDMA: aggravated diarrhea model group with gut microbiota dysbiosis. Compared with the NCD group, * *p* < 0.05, ** *p* < 0.01, *** *p* < 0.001; compared with the HFDM group, ^#^ *p* < 0.05, ^##^ *p* < 0.01.

Targeted UHPLC-MS/MS analysis identified 52 bile acid components in colonic contents from the three groups. The partial least squares-discriminant analysis (PLS-DA) score plot showed an overall separation trend among the NCD, HFDM, and HFDMA groups (Figure 8A). Bile acid composition analysis showed that ω-MCA, DCA, β-MCA, and HDCA were among the predominant bile acid species in all groups (Figure 8B). No significant difference in total bile acid levels was observed among the groups, although both the HFDM and HFDMA groups showed an increasing trend relative to the NCD group (Figure 8C). In contrast, the ratio of secondary to primary bile acids was significantly reduced in the HFDM and HFDMA groups compared with the NCD group, with a more marked decline in the HFDMA group (Figure 8D).

Further screening of differential bile acids using fold change (FC) > 1.2 and *p* < 0.05 identified several characteristic metabolites among groups (Figure 8E). Compared with the NCD group, the HFDM group showed significantly increased CA-7S and 3-DHCA levels, together with significantly decreased 3β-DCA, isoLCA, and β-DHCDCA levels. Compared with the NCD group, the HFDMA group showed significantly decreased 3β-DCA, 3oxo-DCA, IALCA, 7-ketoLCA, HDCA, β-HDCA, and β-DHCDCA levels. Compared with the HFDM group, the HFDMA group showed significantly lower isoLCA and HDCA levels. Overall, the three groups exhibited distinct bile acid profiles in colonic contents, with multiple secondary bile acids reduced, particularly in the HFDMA group.

### 3.7. Correlation Analysis

**Figure 9:**
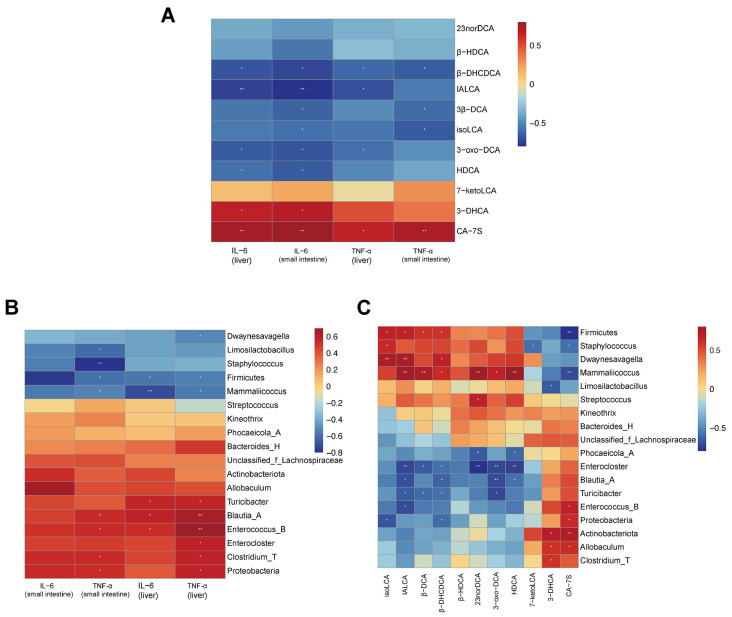
Spearman’s correlation analysis among gut microbiota, differential bile acids, and inflammatory factors in mice. (**A**) Correlation heatmap showing the relationships between differential bile acids and inflammatory factors in the small intestine. (**B**) Correlation heatmap showing the relationships between differential bile acids and inflammatory factors in the liver. (**C**) Correlation heatmap showing the associations among gut microbiota, differential bile acids, and inflammatory factors. Color intensity reflects the strength of the correlation. Red indicates positive correlations, whereas blue indicates negative correlations. * *p* < 0.05, ** *p* < 0.01.

Spearman’s correlation analysis was performed to assess the relationships among gut microbiota, differential bile acids, and inflammatory factors (Figure 9A–C). In both the small intestine and liver, TNF-α and IL-6 were positively correlated with CA-7S and negatively correlated with β-DHCDCA. In the small intestine, both markers were also negatively correlated with isoLCA and 3β-DCA, while IL-6 was further negatively correlated with HDCA, 3-oxo-DCA, and IALCA, but positively correlated with 3-DHCA. In the liver, TNF-α and IL-6 were negatively correlated with 3-oxo-DCA and IALCA, whereas TNF-α was positively correlated with 3-DHCA.

Firmicutes and *Mammaliicoccus* were negatively correlated with hepatic TNF-α and IL-6, as well as with small-intestinal IL-6, whereas *Blautia_A* and *Enterococcus_B* were positively correlated with these inflammatory markers. *Clostridium_T* and Proteobacteria were positively correlated with TNF-α in both tissues. *Dwaynesavagella*, *Enterocloster*, and *Turicibacter* were positively associated with hepatic inflammatory factors, whereas *Limosilactobacillus* and *Staphylococcus* were negatively correlated with small-intestinal TNF-α.

Firmicutes was positively correlated with isoLCA, IALCA, 3β-DCA, and β-DHCDCA, but negatively correlated with CA-7S. Proteobacteria showed positive correlations with isoLCA and β-DHCDCA, but a negative correlation with CA-7S. Actinobacteriota was negatively correlated with 3-DHCA and CA-7S. *Mammaliicoccus* was positively correlated with multiple secondary bile acids but negatively correlated with CA-7S. In contrast, *Enterococcus_B* was positively correlated with CA-7S and negatively correlated with IALCA, whereas *Enterocloster*, *Blautia_A*, and *Turicibacter* were negatively correlated with several secondary bile acids.

Overall, gut microbiota, bile acids, and inflammatory factors were closely associated, supporting the potential involvement of a microbiota–bile acid–inflammation axis in diarrhea.

## 4. Discussion

### 4.1. Bile Acid Metabolism Disorders May Be an Important Factor in Inducing Diarrhea in Mice

When bile acids excessively accumulate in the colon, they can stimulate colonic water and electrolyte secretion and accelerate intestinal transit, thereby inducing diarrhea [20]. Existing studies have shown that bile acid metabolism disorders are present in patients with inflammatory bowel disease, mainly manifested as increased fecal primary and conjugated bile acids together with reduced secondary bile acids [23]. Pediatric patients with Crohn’s disease also exhibit a reduced secondary/primary bile acid ratio [22]. In addition, reduced secondary bile acids have been associated with intestinal inflammatory responses [24]. HFD is considered an important dietary factor capable of disturbing intestinal bile acid homeostasis through both direct effects on bile acid composition and indirect effects mediated by the gut microbiota [8].

In the present study, mice in the HFDM and HFDMA groups showed loose stools, increased fecal water content, reduced locomotor activity, and prolonged immobility time, supporting the successful establishment of the diarrhea model. Consistent with these phenotypic changes, targeted bile acid metabolomics showed that total bile acid levels in colonic contents did not differ significantly among groups, although both model groups showed an upward trend compared with the NCD group. More importantly, the ratio of secondary to primary bile acids was significantly reduced in both the HFDM and HFDMA groups, with a greater reduction observed in the HFDMA group. These findings indicate that HFD-associated diarrhea in this model was accompanied by disturbed bile acid homeostasis, particularly impaired secondary bile acid formation.

Further analysis showed that the HFDM group was characterized by increased CA-7S and 3-DHCA together with decreased 3β-DCA, isoLCA, and β-DHCDCA, whereas the HFDMA group exhibited broader reductions in secondary bile acids, including 3β-DCA, 3-oxo-DCA, IALCA, 7-ketoLCA, HDCA, β-HDCA, and β-DHCDCA. Previous evidence indicates that several of these bile acids are linked to intestinal immune homeostasis and barrier maintenance. Notably, 3-oxo-DCA may exert anti-inflammatory effects by inhibiting M1 macrophage activity and reducing the secretion of IL-1β and TNF-α [25]. isoLCA inhibits T helper 17 (Th17) cell differentiation by directly binding to retinoic acid receptor-related orphan receptor gamma t (RORγt) [24] and can also upregulate ZO-1 and Claudin-1 expression to alleviate dextran sulfate sodium (DSS)-induced colonic barrier injury [26]. Microbial-derived bile acid epimers, including iLCA and iaLCA, have been reported to inhibit *Clostridioides difficile* growth and pathogenicity in vitro [26]. In addition, 7-ketoLCA can alleviate aspirin-induced intestinal injury [27], and HDCA has been shown to reduce TNF-α and IL-6 levels [28]. In our study, TNF-α and IL-6 levels in the small intestine and liver were negatively correlated with several secondary bile acids, but positively correlated with CA-7S. Taken together, these findings support an association between bile acid metabolic disturbance, especially reduced secondary bile acids, and intestine–liver inflammatory responses in diarrhea. However, these results should be interpreted as evidence of association rather than direct causation.

### 4.2. Bile Acid Metabolic Dysregulation in Mice with Diarrhea May Be Associated with Intestinal Microbiota Dysbiosis

The gut microbiota is essential for host health, and disruption of its structure is widely considered a defining feature of diarrhea [29,30]. The interaction between the gut microbiota and the host is highly dynamic and complex, and microbial metabolites play a central mediating role in this crosstalk [31]. Previous studies have shown that dysbiosis of the gut microbiota may facilitate diarrhea by disturbing bile acid metabolism [29,32]. In the present study, small-intestinal contents were used for microbiota profiling because the small intestine is the main site of nutrient digestion and absorption and plays an important role in host–microbe interactions, immune regulation, and bile acid homeostasis [33]. Moreover, the major barrier injury in this model was observed in the small intestine, making small-intestinal contents more relevant to the local pathological changes.

In our study, α-diversity indices in both the HFDM and HFDMA groups showed downward trends relative to the NCD group, and the HFDMA group tended to show lower values than the HFDM group, although these differences were not statistically significant. β-diversity analysis also showed a separation trend among groups. Therefore, the present results support altered microbial community structure in diarrheic mice but do not support overstatement of a statistically stronger dysbiosis in the HFDMA group based on diversity indices alone.

At the compositional level, both model groups showed an increasing trend in Proteobacteria, whereas *Ligilactobacillus* showed a decreasing trend and was significantly reduced in the HFDMA group. The proliferation of Proteobacteria is commonly regarded as a feature of microbiota dysbiosis and is closely associated with intestinal inflammation [32]. As a beneficial commensal, *Ligilactobacillus* murinus can modulate macrophage-mediated anti-inflammatory responses and help maintain intestinal barrier integrity [33,34]. Therefore, the decrease in *Ligilactobacillus* in diarrheic mice may be relevant to the reduced expression of ZO-1 and Claudin-1 observed in this study.

LEfSe and random forest analyses further identified Enterocloster, Turicibacter, Streptococcus, Kineothrix, Blautia_A, Mammaliicoccus, Limosilactobacillus, and Enterococcus_B as representative taxa contributing to group discrimination. PICRUSt2-based functional prediction showed that secondary bile acid biosynthesis displayed a downward trend in the HFDM group and a lower predicted abundance in the HFDMA group than in the NCD group, suggesting that HFD-associated microbial restructuring was accompanied by altered bile acid-related metabolic potential. HFD has been linked in prior work to gut microbiome dysbiosis, bile acid disorders, and intestinal inflammation, which is consistent with the overall pattern observed here [8]. This interpretation is further supported by recent nutrition research showing that overall dietary composition is closely related to gut microbiota profiles, with both beneficial and unfavorable dietary components shaping the gut microbiota [35,36,37].

Among the bacteria identified in this study, the relationship between Firmicutes-related taxa and bile acid metabolism appears particularly relevant. Several secondary bile acids, including isoLCA, IALCA, 3β-DCA, and β-DHCDCA, were positively correlated with Firmicutes-related taxa, whereas CA-7S showed a negative correlation. This is largely consistent with previous research findings [29] and suggests an association between depletion of Firmicutes-related bacteria and reduced secondary bile acid formation. Likewise, the decrease in *Limosilactobacillus*/*Ligilactobacillus* may also be meaningful because *Lactobacillus*-related taxa are often involved in the upstream deconjugation step of microbial bile acid transformation through bile salt hydrolase activity [38].

In contrast, *Enterococcus_B* was negatively correlated with several secondary bile acids and positively correlated with CA-7S, while *Turicibacter* and *Enterocloster* were negatively correlated with multiple secondary bile acids. These findings are noteworthy because *Enterococcus* species possess active bile salt hydrolases and strong bile tolerance [39,40], whereas recent evidence indicates that *Turicibacter* strains can directly modify host bile acids in a strain-dependent manner and thereby reshape host bile acid profiles [41]. Overall, these findings suggest that HFD-related microbial alterations were closely associated with bile acid dysregulation in diarrheic mice. However, because these observations are based on correlation analysis and predictive functional analysis, they should still be interpreted as evidence of association rather than direct causation.

Analysis of the relationships between gut microbiota and inflammatory factors indicated that Firmicutes-related taxa and *Mammallicoccus* were negatively correlated with hepatic TNF-α and IL-6 and with IL-6 in the small intestine. By contrast, *Blautia_A*, *Enterococcus_B*, *Clostridium_T*, Proteobacteria, *Enterocloster*, and *Turicibacter* were positively correlated with selected inflammatory parameters.

### 4.3. Inflammatory Mediators Are Involved in the Gut–Liver Interaction Injury Observed in Mice with Diarrhea

Increasing evidence in recent years has highlighted the gut–liver axis as a critical perspective for understanding diarrhea development [9,42]. In this axis, bile acids are essential for immune regulation and maintenance of the intestinal barrier. Barrier disruption can increase intestinal permeability, facilitating the translocation of bacterial endotoxins, including LPS, into the portal vein and contributing to liver inflammation and damage [41,42]. Conversely, hepatic inflammation can further aggravate intestinal inflammation and compromise barrier integrity through the release of cytokines and chemokines, thus forming a deleterious feedback loop along the gut–liver axis. Current work also supports the view that HFD can impair intestinal barrier integrity and enhance gut-derived inflammatory signaling, thereby contributing to intestine–liver injury [8,41,42,43].

IL-6 and TNF-α are key inflammatory mediators in this process. In addition to mediating inflammatory responses, they can also increase intestinal permeability by disrupting tight junction complexes [44].

In the present study, both the HFDM and HFDMA groups showed clear histopathological injury in the small intestine and liver, accompanied by significantly elevated IL-6 and TNF-α levels in both tissues. At the same time, ZO-1 and Claudin-1 expression in the small intestine was reduced in the two model groups. These findings support the presence of concurrent barrier impairment and intestine–liver inflammatory injury in mice exposed to fatigue combined with HFD. However, although histological injury appeared more pronounced in the HFDMA group, inflammatory cytokine levels and barrier protein expression did not differ significantly between the HFDM and HFDMA groups. Therefore, the present data support the presence of HFD-related intestine–liver injury in this model but do not prove a stronger inflammatory response in the HFDMA group at the molecular level.

This study has some limitations. It was conducted in a mouse model of fatigue- and HFD-induced diarrhea, and its applicability to human disease still needs further validation. In addition, although alterations were observed in the gut microbiota, bile acid profiles, barrier function, and intestine–liver inflammatory responses, the causal links among these changes remain to be further elucidated. In addition, small-intestinal contents were used for microbiota profiling, whereas colonic contents were used for bile acid analysis. Although this sampling strategy was biologically justified, it may have limited the direct correspondence between microbial alterations and bile acid changes. Moreover, no positive control group was included because the primary aim of this study was mechanistic exploration rather than efficacy evaluation. Future studies incorporating direct functional validation, positive control design, and human data are warranted to further clarify the causal links among HFD, gut microbiota, bile acid metabolism, and intestine–liver injury in diarrhea. A positive control was not included because this study was designed primarily as a mechanistic study rather than a therapeutic intervention study. The antibiotic-treated HFDMA group was used to aggravate gut microbiota dysbiosis. Therefore, the absence of a positive control should be considered a limitation.

## 5. Conclusions

In summary, fatigue combined with HFD-induced diarrhea accompanied by gut microbiota dysbiosis, bile acid metabolic imbalance, intestinal barrier damage, and elevated inflammatory responses in the small intestine and liver. These alterations may act synergistically through the gut–liver axis to promote disease progression. The present findings expand current understanding of the underlying mechanisms and provide a theoretical foundation for future mechanistic and therapeutic studies.

## Data Availability

The gut microbiome sequencing data have been uploaded to the NCBI database (https://www.ncbi.nlm.nih.gov/, accessed on 22 December 2025), no. PRJNA1201605.

## Supplementary Materials

The following supporting information can be downloaded at https://www.mdpi.com/article/10.3390/nu18091317/s1. Supplementary Material S1: Detailed per-sample sequencing statistics, including Q20, Q30, GC content, and merged-read counts. Supplementary Material S2: Detailed validation data of UHPLC-MS/MS Analysis.
